# Application of Telehealth in Prenatal Care during the COVID-19 Pandemic—A Cross-Sectional Survey of Polish Women

**DOI:** 10.3390/jcm10122570

**Published:** 2021-06-10

**Authors:** Dominik Jakubowski, Dorota Sys, Anna Kajdy, Roksana Lewandowska, Ewa Kwiatkowska, Aneta Cymbaluk-Płoska, Michał Rabijewski, Andrzej Torbé, Sebastian Kwiatkowski

**Affiliations:** 1 Department Obstetrics and Gynecology, Pomeranian Medical University, Al. Powstańców Wlkp. 72, 70-111 Szczecin, Poland; ddominikjakubowski@gmail.com (D.J.); roksanna.lew@gmail.com (R.L.); torbea@wp.pl (A.T.); kwiatkowskiseba@gmail.com (S.K.); 2 Department of Reproductive Health, Centre of Postgraduate Medical Education, Żelazna 90 St., 01-004 Warsaw, Poland; dsys@cmkp.edu.pl (D.S.); mirab@cmkp.edu.pl (M.R.); 3 Department of Nephrology, Transplantology and Internal Medicine, Pomeranian Medical University, Al. Powstańców Wlkp. 72, 70-111 Szczecin, Poland; ewakwiat@gmail.com; 4 Department of Gynecological Surgery and Gynecological Oncology of Adults and Adolescents, Pomeranian Medical University, Al. Powstańców Wlkp. 72, 70-111 Szczecin, Poland; aneta.cymbaluk@gmail.com

**Keywords:** SARS-CoV-2, COVID-19, prenatal care, standard of care, telemedicine, cross-sectional studies, Poland

## Abstract

To reduce the risk of infection of SARS-CoV-2 during commuting to the clinic or due to contact with medical staff, the American College of Obstetricians and Gynecologists has recommended arranging some appointments in the form of “telehealth”. The aim of the study was to assess the access to medical care in pregnancy during the SARS-CoV-2 pandemic and the role of telehealth in the implementation of prenatal care standards. This is a cross-sectional study. The study group included 618 women who were pregnant and/or gave birth in Poland during the COVID-19 pandemic. The majority of the participants experienced difficulties accessing medical care because of the pandemic. The correlation between this experience and the use of the hybrid healthcare model was established. The affiliation to public or private healthcare was irrelevant. There was no relationship between healthcare (private/public or in-person/hybrid) and implementation of the prenatal care standards. To ensure safe access to prenatal care for pregnant women, recommendations for a hybrid pregnancy management model should be created with detailed information regarding which appointments patients must be present for in-person and which can be conducted remotely. To reduce the risks associated with movement and interpersonal contact, all visits during which tests and screenings take place should be conducted in-person; other appointments can be arranged in the form of telehealth.

## 1. Introduction

Severe acute respiratory syndrome coronavirus 2 (SARS-CoV-2) was first identified in December 2019 in the city of Wuhan in China. The virus quickly started to spread all over the world [1]. On 24 January 2020, the first case in Europe was diagnosed, and on 4 March 2020, in Poland. On 30 January 2020, the World Health Organization (WHO) classified COVID-19 as a threat to public health. On 11 March 2020, a world pandemic was declared [2]. The most commonly reported symptoms are a cough, dyspnea, and fever. It has not been proven that infection increases the risk of antenatal complications [3]. The risk of vertical infection, fetal growth restriction, miscarriage, and preterm birth is still widely debated [4]. The latest systematic review of pregnancy outcomes provides more insight into the risk of transmitting the infection from mother to child [5].

Countries across the world have initiated procedures to prevent the spread of the virus and the development of severe disease. Due to the lack of a treatment regimen and data regarding long-term complications of the disease, planned hospital admissions and doctor appointments were curtailed. Pregnant women were put in a challenging position where they had to follow a strict management plan of scheduled visits with their obstetrician or midwife during their pregnancy [6]. To reduce the risk of infection during their commute to the clinic or due to contact with medical staff, the American College of Obstetricians and Gynecologists (ACOG) recommended arranging some appointments in the form of “telehealth” [7]. This term is defined as delivering remote medical services by healthcare providers using technology to communicate with patients to diagnose, treat, and educate them on topics regarding their health [8]. Appointments are most often conducted by video chat, but they can also be conducted by phone when there is no access to a camera. Additionally, portable devices such as sphygmomanometers, glucometers, pulse oximeters, and mobile CTG devices are implemented to monitor patient wellbeing [9]. In a study by Futterman et al., slightly lower satisfaction scores were recorded for telehealth prenatal appointments in comparison to in-person appointments, but the study did not yield clinically significant differences in their outcomes [10].

In Poland, pregnant patients have medical appointments scheduled every 4 weeks and, after 34 weeks of gestation, every 2 weeks [11]. An essential part of antenatal care is an ultrasound screening in the first, second, and third trimesters at 11–13.6 weeks, 18–22 weeks, and 28–32 weeks, respectively. The purpose of this screening is to check for chromosomal anomalies, perform an anatomy assessment, and conduct a growth scan. According to the Polish Society of Gynecologists and Obstetricians’ recommendations, the critical aspect of antenatal care is to perform ultrasound screenings within the recommended timeframe [12]. The WHO ANC Model from 2016 recommends eight medical visits during pregnancy [12,13]. The number of prenatal appointments can vary depending on the condition of both the mother and her child.

The aim of the study was to assess the access to medical care in pregnancy during the SARS-CoV-2 pandemic and the role of telehealth in the implementation of prenatal care standards.

## 2. Materials and Methods

### 2.1. Study Design

This is a cross-sectional study. The study group included women who were pregnant and/or gave birth in Poland during the COVID-19 pandemic. It is a convenience sample, meaning that every woman who received information about this study and consented to participate could participate. At the beginning of the questionnaire, participants were informed about the aim of the study, the time needed to complete the survey, ways in which the gathered data would be used, and the fact that completing the survey was considered as giving consent for participation. The questionnaire was anonymous, and no information that could identify the respondents was collected. Because of the anonymity of the questionnaire, it was impossible to withdraw submitted answers.

The survey was prepared by specialists in the field of obstetrics, gynecology, and perinatology. It was divided into five parts:

- Questions about demographics, chronic diseases, access to medical care during the pandemic, exposure to COVID-19 infection;
- Questions about the course of their pregnancy, screening tests, ultrasounds, and antenatal medical appointments with their obstetrician;
- Questions about the laboratory tests performed during their pregnancy;
- Questions about parturition and the child’s condition;
- Questions about mental state and any miscarriages.

After conducting a pilot study on a group of women, experts assessed the relevance of this questionnaire and the notes from participants and proposed their questions based on these assessments. The final survey consisted of 98 questions in Polish.

The study data were collected and managed using REDCap electronic data capture tools hosted at The Foundation of St. Sophia’s Specialist Hospital. REDCap (Research Electronic Data Capture) is a secure, web-based software platform designed to support data capture for research studies, including questionnaire surveys [14,15]. The survey was an open survey, available to every internet user with no need for registration or logging in. The link to the survey was shared on social media, particularly on groups dedicated to pregnancy and on the prenatalproject.org and szpitalzelazna.pl websites. The survey was voluntary, and participants did not receive any reward or “presents” for completing the questionnaire. Data were collected from 5 August 2020 to 29 October 2020.

The questionnaire was divided into five viewscreens by the topics of the questions. Every question was mandatory (where it was justified, the answer “not applicable” or “rather not say” was available). The completeness and correctness of the responses were checked each time before participants were allowed to proceed to the next viewscreen. In the case of incompleteness or incorrectness in the questionnaire, the website displayed a message asking the participant to correct their responses. Participants were able to go back to previous viewscreens to check and change their answers.

The website was viewed 9119 times by internet users. A total of 1351 people participated in the study and completed the screening questions. The recruitment rate was 15%. A total of 1312 participants completed the questionnaire in full, and the completeness rate was 97%.

### 2.2. Reporting, Ethics, and Dissemination

The STROBE and Cherries guidelines were used to ensure proper reporting of this cross-sectional web-based survey [16,17]. The study was conducted according to the criteria set by the Declaration of Helsinki. Surveys as noninterventional studies do not require assessment by a bioethics committee according to Polish research law. The respondents were informed that the survey was anonymous. The survey consisted of an information letter and a statement which explained that, by filling out and returning the survey, the participant was giving their informed consent to participate in the study.

### 2.3. Statistical Analysis

Statistical Package for Social Sciences (SPSS) for Mac, version 27.0.0.0 (IBM Corp., Armonk, NY, USA) was used to analyze statistical data. The count variables were presented as medians. To compare quantitative variables, the Mann–Whitney test was used. To compare the dichotomous variables, the chi-square test was used. Realization of the prenatal care standard was compared with the hybrid and in-person healthcare groups and then between the private and public healthcare groups using the chi-square test. The statistical significance level was defined at α < 0.05.

## 3. Results

The web-based survey yielded 1312 individual responses. Respondents that were not pregnant during the COVID-19 pandemic (*n* = 425) were excluded from the study. After accessing the histograms looking for outliers, only participants between the ages of 18 and 40 were included in the study. Additionally, the following inclusion criteria were defined: declaration of being pregnant or giving birth during the COVID-19 pandemic, filling in the questionnaire in Polish, and answering all the mandatory questions. The exclusion criteria included respondents aged below 18 or above 40 and those who were not pregnant and/or gave birth during the COVID-19 pandemic. In the end, the number of respondents in the study group was 618 patients. The characteristics of the study group are presented in Table 1. The respondents declared their state of residence, and this was divided depending on the number of inhabitants (32% > 500,000, 40% 100,00–500,000, 28% < 10,000). Patients were divided into groups by the type of appointments they had during their pregnancy. The first group (*n* = 293) had in-person appointments and at least one telehealth appointment during pregnancy, while the second group (*n* = 325) had only in-person appointments. Patients were also divided by their declared access to private (*n* = 477) or public (*n* = 141) healthcare.

In the next stage of the study, it was determined which patients had the prenatal care standard implemented, e.g., had all the tests and screenings for their gestational age as recommended in Poland. In this step, all the patients who had not finished their first trimester were excluded from the study as it was not possible to assess whether the standard was fully implemented in such an early stage of pregnancy. Therefore, the implementation of the prenatal care standard was evaluated in 550 women (*n* = 550).

A Mann–Whitney test showed that the women who had access to private medical care were older (Mdn = 30) in comparison to the women who accessed public healthcare (Mdn = 29), U = 28.14, *p* = 0.003. A quantitative variables comparison showed no statistically significant differences between the in-person healthcare group and the hybrid healthcare group.

The chi-square test was conducted to check the hypothesis that healthcare (in-person or hybrid) is linked with sufficient access to tests and screening procedures during pregnancy. In the access to the combined screening test, there was a statistically significant difference between the study groups X^2^ (1, N = 618) = 4.83, *p* = 0.017, and Cramer’s V = 0.09. Patients who only had in-person visits had an OGTT less often than those who had at least one remote appointment X^2^ (1, N = 618) = 3.68, *p* = 0.055, and Cramer’s V = 0.07. A significant difference between the study groups was also found regarding ultrasound screenings between 28 and 32 weeks of gestation X^2^ (1, N = 618) = 7.51, *p* = 0.006, and Cramer’s V = 0.11, as well as regarding having experienced difficulties accessing prenatal care because of the COVID-19 pandemic X^2^ (1, N = 618) = 29.14, *p* < 0.001, and Cramer’s V = 0.22. Detailed information can be found in Table 2.

A chi-square test was conducted to assess the relationship between the private and public healthcare groups and the access to tests and screenings during pregnancy. Significant statistical differences were found between the groups regarding access to fasting glucose tests X^2^ (1, N = 618) = 8.807, *p* = 0.003, and Cramer’s V = 0.12, oral glucose tolerance tests (OGTT) X^2^ (1, N = 618) = 5.28, *p* = 0.022, and Cramer’s V = 0.09, and ultrasound screening between 28 and 32 weeks of gestation X^2^ (1, N = 618) = 9.28, *p* = 0.002, and Cramer’s V = 0.12. Detailed information can be found in Table 3.

A chi-square test was conducted to assess the relationship between the type of healthcare and the implementation of the prenatal care standards, i.e., all tests and screenings recommended for the gestational age. No statistically significant difference was found between the in-person and hybrid healthcare groups, X^2^ (1, N = 550) = 0.103, *p* = 0.748, and Cramer’s V = 0.01. Detailed information can be found in Table 4. In the private and public healthcare groups, there was no statistically significant difference, X^2^ (1, N = 550) = 0.272, *p* = 0.342, and Cramer’s V = 0.064. Detailed information regarding these groups can be found in Table 5.

A chi-square test was conducted to assess the relationship between the use of public or private healthcare and hybrid or in-person pregnancy management types. There was no statistically significant difference found, X^2^ (1, N = 618) = 0.126 and *p* = 0.398.

## 4. Discussion

The majority of participants experienced difficulties in accessing medical care because of the pandemic. A correlation between this experience and the use of the hybrid healthcare model was established. The affiliation to the public or private healthcare group was irrelevant. There was no relationship between healthcare (private/public or in-person/hybrid) and implementation of the prenatal care standards. Before the pandemic, telehealth was not routinely used in pregnancy management. Only one paper can be found in the literature regarding the use of telehealth in obstetrics before 2020. Karwowski et al. showed that in Poland, most patients sought telehealth medical help in cases of potential abortions or premature birth [18]. Our research demonstrated that 47.41% of women had at least one telehealth appointment during their pregnancy. This number is higher compared to the research conducted by Madden et al., where the percentage of telehealth appointments in New York during the COVID-19 pandemic was 31.8% [19]. In the ASPE (Assistant Secretary for Planning and Evaluation) report, the use of telehealth in the biggest cities in the United States increased from 0.1% in February 2020 to 43.5% in April 2020 [18,20].

Telehealth was used to a similar extent in public (46.01%) and private (48%) healthcare in this study. In comparison, in Australia in 2014, 68% of the healthcare providers that offered telehealth services were a part of the public healthcare system [21]. In a meta-analysis by Xie et al., it was reported that patients using telehealth controlled their blood glucose levels better and had a lower risk of maternal and fetal complications than those in the in-person group [22].

According to the Royal College of Obstetricians and Gynaecologists’ recommendations, it was better to do an HbA1c test instead of an OGTT during the pandemic restrictions [23]. A meta-analysis showed that women who had telehealth medical appointments had lower glycosylated hemoglobin levels than those who only had in-person visits [24,25]. According to the Polish Diabetes Society’s guidelines, gestational diabetes mellitus (GDM) is diagnosed with a fasting glucose test and an OGTT [26]. Our research showed a statistically significant difference in access to those tests in the private and public healthcare groups in favor of private healthcare. Siru et al. demonstrated that the sole use of a fasting glucose test without following with an OGTT can result in more undiagnosed cases of gestational diabetes (GDM) [26].

Our research showed that primigravida women attended in-person appointments more often than hybrid prenatal appointments. These results contradict those obtained by Du et al., according to whom primigravida women prefer telehealth visits. It is possible that our participants chose the in-person model because of the fear of prenatal complications [27].

Only 49.45% of the participants had prenatal healthcare standards fully implemented regardless of their affiliation to the in-person or hybrid healthcare model groups or their use of private or public medical care. Interestingly, a relationship was found between being in the hybrid healthcare model group and experiencing difficulties in accessing medical care because of the pandemic, even though the rate of prenatal healthcare standard implementation in both groups was similar. It was shown that 73.14% of women experienced difficulties accessing medical care during the COVID-19 pandemic. In comparison, in survey research conducted by Ceulemans et al., 61.8% of women received less medical help from their obstetrician after the COVID-19 pandemic than they did before [28].

Another interesting finding is that patients using private healthcare were more likely to have had a fasting glucose test, an OGTT, and an ultrasound screening between 28 and 32 weeks of gestation than those using public healthcare. This is probably related to the fact that patients canceled their appointments in medical facilities because they feared contracting SARS-CoV-2. Justmana et al. reported a lower number of admissions to obstetrics wards and ultrasound screenings in March–April 2020 compared to the same period in the preceding year [29]. Another analysis showed that almost half of the participants considered canceling their prenatal appointments in the hospital due to the pandemic. At the same time, 20% of patients experienced anxiety associated with any visit to a medical facility [30]. Because of movement restrictions implemented due to the pandemic, it seems beneficial to recommend a hybrid healthcare model. Appointments during which patients have tests and screenings should be conducted in-person, while follow-up visits can be carried out via telehealth. This model allows us to lower the risk of COVID-19 infection while maintaining a high prenatal care standard. In this context, the hybrid prenatal care model seems more beneficial than the traditional in-person care model.

This study has several strengths: It was conducted in a fairly homogenous population of Polish women. The questions asked in the survey assessed both objective and subjective access to prenatal care during the pandemic. In-person and hybrid healthcare models were predefined. Access and implementation of perinatal care was defined according to national standards. On the other hand, the study does also have limitations: The questionnaire surveys shared online were at risk of bias, including selection bias, non-response bias, response bias, recall bias, and attentional bias. The conducted statistical analyses could be impaired by omitted-variable bias.

Standards of perinatal care vary worldwide, but the mere necessity of prenatal care is universal, and telehealth solutions can be implemented in all developed countries.

## 5. Conclusions

We are currently experiencing the third wave of the COVID-19 pandemic. To ensure safe access to prenatal care for pregnant women, recommendations for a hybrid pregnancy management model should be created with detailed information for which appointments patients must be present for in-person and which can be conducted remotely. To reduce the risks associated with movement and interpersonal contact, all appointments during which tests and screenings take place should be conducted in-person; other appointments can be arranged in the form of telehealth.

## Figures and Tables

**Table 1:** Characteristics of the study group.

Variable	Yes	% Yes
Birth during COVID-19 pandemic	246	39.8%
Experiencing difficulties accessing medical care because of pandemic.	452	73.14%
Changing obstetrician because of pandemic.	74	11.97%
Having a telehealth prenatal appointment during pandemic.	293	47.41%
Being tested for COVID-19 during pregnancy.	63	10.19%
Being quarantined during COVID-19 pandemic.	17	2.75%
Primiparity.	351	56.8%
Ultrasound before 10 weeks of gestation.	578	93.53%
Ultrasound screening between 11 and 13 + 6 weeks of gestation.	603	97.57%
Combined screening test.	364	58.9%
Ultrasound screening between 18 and 22 weeks of gestation.	555	89.8%
Fasting glucose test.	574	92.88%
Oral glucose tolerance test.	470	76.05%
Ultrasound screening between 28 and 32 weeks of gestation.	420	67.96%
Diagnosed gestational diabetes mellitus (GDM).	99	16.02%
Diagnosed anemia during pregnancy.	105	16.99%
Access to private healthcare.	477	77.18%

**Table 2:** Comparison of logical variables between in-person and hybrid healthcare groups.

	Group of Hybrid Healthcare		Group of In-Person Healthcare		*p*-Value
Variable	Yes	% Yes	Yes	% Yes	
Experiencing difficulties accessing medical care because of pandemic.	244	83.28%	208	64%	<0.001
Primogeniture.	126	43%	225	69.23%	<0.001
Diagnosed gestational diabetes mellitus (GDM).	55	18.77%	44	13.54%	0.077
Diagnosed hypothyroidism during pregnancy.	86	29.35%	71	21.85%	0.032
Diagnosed anemia during pregnancy.	52	17.75%	53	16.31%	0.634
Ultrasound before 10 weeks of gestation.	276	94.2%	302	92.92%	0.520
Ultrasound screening between 11 and 13 + 6 weeks of gestation.	285	97.27%	318	97.85%	0.642
Double marker test.	186	64.48%	178	54.77%	0.028
Ultrasound screening between 18 and 22 weeks of gestation.	269	91.81%	286	88%	0.118
Fasting glucose test.	273	93.17%	301	92.61%	0.787
OGTT.	233	79.52%	237	72.92%	0.055
Ultrasound screening between 28 and 32 weeks of gestation.	215	73.38%	205	63.08%	0.006

**Table 3:** Comparison of logical variables between private and public healthcare groups.

	Private Health Group		Public Health Group		*p*-Value
Variable	Yes	% Yes	Yes	% Yes	
Experiencing difficulties accessing medical care because of pandemic.	344	72.12%	108	76.6%	0.172
Diagnosed gestational diabetes mellitus (GDM).	79	16.56%	20	14.18%	0.297
Diagnosed anemia during pregnancy.	79	16.56%	26	18.44%	0.342
Ultrasound before 10 weeks of gestation.	450	94.34%	128	90.78%	0.097
Ultrasound screening between 11 and 13 + 6 weeks of gestation.	467	97.9%	136	96.45%	0.242
Double marker test.	289	60.59%	75	53.19%	0.117
Ultrasound screening between 18 and 22 weeks of gestation.	432	90.57%	123	87.23%	0.251
Fasting glucose test.	451	94.55%	123	87.23%	0.003
OGTT.	373	78.2%	97	68.79%	0.022
Ultrasound screening between 28 and 32 weeks of gestation.	339	71.07%	81	57.45%	0.002

**Table 4:** Characteristics of prenatal care standard implementation in hybrid and in-person healthcare groups.

		Hybrid Healthcare		In-Person Healthcare		In total	
		N	%	N	%	N	%
Completely implemented standard	no	127	50.2%	145	48.8%	272	49.5%
	yes	126	49.8%	152	51.2%	278	50.5%
In total		253	100%	297	100%	550	100%

**Table 5:** Characteristics of prenatal care standard implementation in public and private healthcare groups.

		Public Healthcare		Private Healthcare		In total	
		N	%	N	%	N	%
Completely implemented standard	no	62	55.9%	210	47.8%	272	49.5%
	yes	49	44.1%	229	52.2%	278	50.5%
In total		111	100%	439	100%	550	100%

## Data Availability

The data presented in this study are available on request from the corresponding author.
